# Opportunistic osteoporosis screening via the measurement of frontal skull Hounsfield units derived from brain computed tomography images

**DOI:** 10.1371/journal.pone.0197336

**Published:** 2018-05-10

**Authors:** Min Kyun Na, Yu Deok Won, Choong Hyun Kim, Jae Min Kim, Jin Hwan Cheong, Je Il Ryu, Myung-Hoon Han

**Affiliations:** Department of Neurosurgery, Hanyang University Guri Hospital, Korea; Universidad de Zaragoza, SPAIN

## Abstract

**Background and purpose:**

Osteoporosis is one of the most common chronic metabolic diseases, but detection and treatment rates are low. The aim of the current study was to evaluate the correlation between frontal skull Hounsfield unit (HU) values from brain computed tomography (CT) scans and T-scores of the lumbar spine and femoral neck from dual-energy X-ray absorptiometry (DXA) scans.

**Methods:**

Patients with < 1 year between brain CT and DXA scans were included in the study. The average frontal skull HU value used for analysis was defined as the average of four HU values of the frontal bone. A receiver operating characteristic curve was generated, and area under the curve (AUC) was used to determine the HU values of the frontal skull for predicting osteoporosis. The frontal skull HU value with the highest sensitivity and specificity was considered the optimal cutoff value.

**Results:**

In total, 899 patients who underwent both brain CT and DXA scans at a single institution were enrolled. Average skull HU values differed significantly among patients in different bone mineral density categories (*p* < 0.001). There was a positive correlation between skull HU value and T-score (β = 105.06, *p* < 0.001, R^2^ = 0.343). The mean HU value in subjects with osteoporosis was 515, and the optimal cutoff value for the prediction of osteoporosis was 610 HU (AUC = 0.775, 95% CI 0.744–0.806, *p* < 0.001).

**Conclusions:**

Clinical brain CT scans can assist in the detection of osteoporosis, and patients with an HU value < 610 as determined via brain CT may be considered for further evaluation for possible osteoporosis.

## Introduction

Osteoporosis is one of the most common chronic metabolic diseases, and it is characterized by reduced bone mineral density, altered non-collagenous proteins, disrupted bone microarchitecture, higher bone fragility, and an increased fracture risk [1]. Estimates suggest that 125 million people in Europe, India, Japan, and the USA meet the criteria for osteoporosis, and that 1 in 3 women and 1 in 5 men over the age of 50 will experience an osteoporotic fracture [2]. The incidence of osteoporotic fragile fractures is expected to increase 2 to 4-fold within the next 30 years, and healthcare costs associated with osteoporosis will continue to increase [3–5]. Despite the proven efficacy and cost-effectiveness of osteoporosis diagnosis and treatment, detection and treatment rates remain low [6]. A previous study showed that less than 10% of patients with distal radial fracture underwent appropriate diagnosis and medical treatment for osteoporosis, suggesting that surgeons need to identify high-risk patients who require active screening for osteoporosis at the time of fracture [7]. To overcome the low rates of dual-energy X-ray absorptiometry (DXA) screening, previous studies have aimed to find adequate screening tools for osteoporosis during non-specific evaluations and have proposed the use of attenuation data from clinical computed tomography (CT) scans [6].

In the current study, we aimed to evaluate the correlation between frontal skull Hounsfield unit (HU) values measured via brain CT, and T-scores of the lumbar spine and femoral neck derived from DXA scans. We also sought to identify a threshold skull HU value for the prediction of osteoporosis.

## Methods

### Patient selection

We retrospectively extracted data from patients aged > 18 years with one or more procedure codes for DXA and brain CT scans among all patients who visited or were admitted to Hanyang University Guri Hospital, Korea, from 1 January 2010 to 31 December 2016. Initially, a total of 1825 patients who underwent at least one DXA scan and one brain CT scan were identified. In patients who underwent multiple DXA scans, we used their lowest T-score. A brain CT was then selected for analysis. In patients who received multiple CT scans, we selected the one performed closest to the date of the selected DXA scan. To reduce time heterogeneity, we then excluded 894 patients in which there had been > 1 year between DXA and brain CT scans (S1 Fig). We also excluded a further 32 patients who showed no measurable cancellous bone of the frontal skull (too narrow a space between both cortical bones). These 926 exclusions resulted in a final study sample of 899 patients.

This study was approved by the Institutional Review Board of Hanyang University Guri Hospital, Korea, and conformed to the tenets of the Declaration of Helsinki. Owing to the retrospective nature of the study, the need for informed consent was waived. All patient records were anonymized prior to analysis.

### Bone mineral density measurement

DXA to assess the bone mineral density (BMD, g/cm^2^) of the lumbar spine L1–L4 and femoral neck was performed using the Discovery Wi DXA system (Hologic, Bedford, MA) in all patients. All testing was conducted by licensed technicians. The BMD values were converted into a T-score. T-score reference ranges were calculated using values derived from healthy young Asian female and male subjects that were provided by a bone densitometry manufacturer [8]. T-score was defined as the BMD of participant − mean BMD of the reference population/standard deviation (SD) of the reference population [9]. Each patient’s BMD was categorized as normal, osteopenic, or osteoporotic based on the World Health Organization T-score classifications, where osteoporosis is defined as a T-score ≤ −2.5, osteopenia is defined as a T-score > −2.5 and ≤ −1.0, and normal BMD is defined as a T-score > −1.0. The lower T-score of those of the lumbar spine and femoral neck was used as the T-score in the study.

### Measurement of skull HU

**Fig 1:**
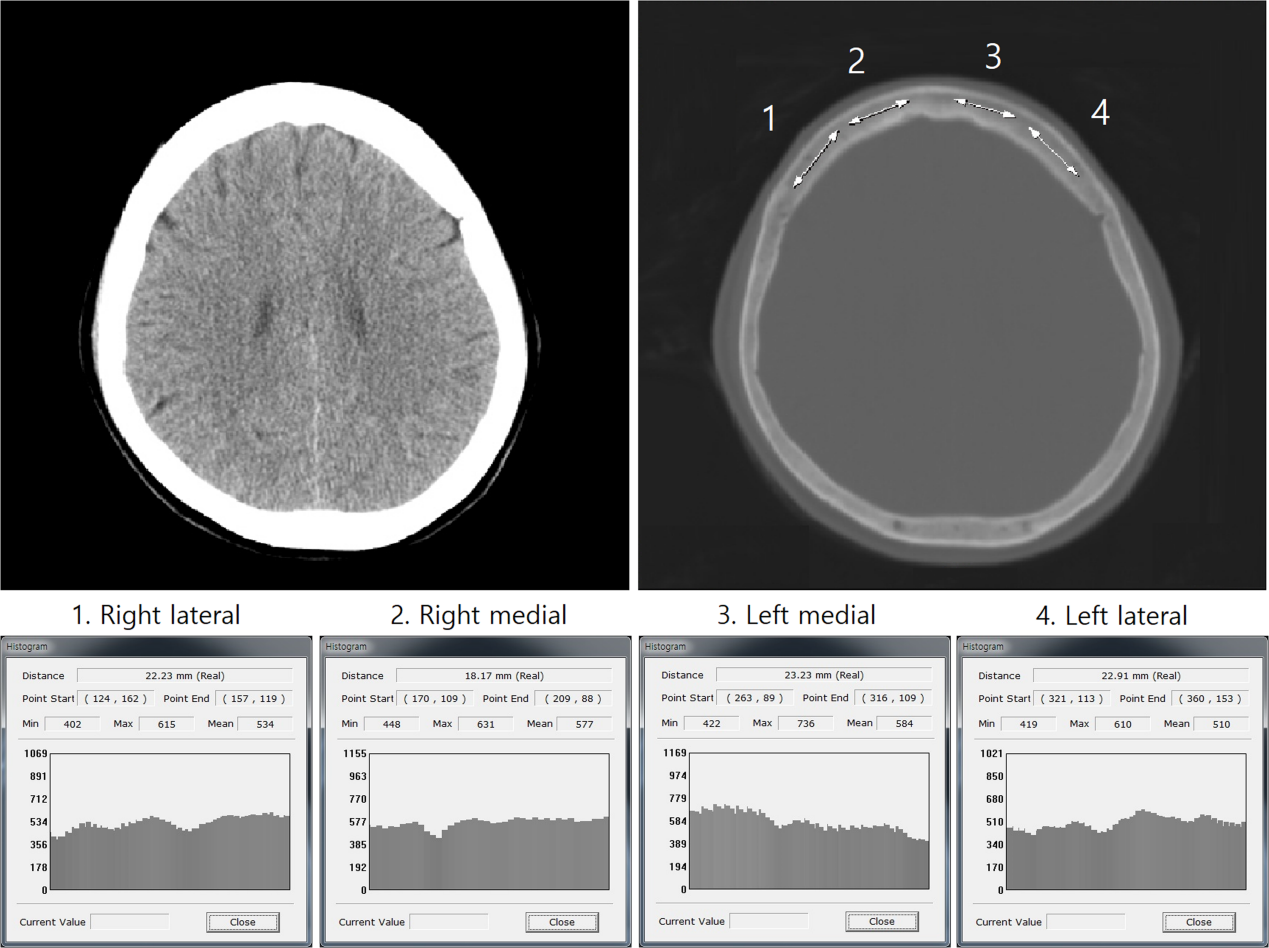
The average HU value of each of the four lines on the frontal bone. The PACS automatically calculates and provides maximum, minimum, and average HU values according to the drawing line.

All CT images (4.0–5.0-mm slice thicknesses, 100 kVp) were obtained with a CT scanner (Siemens Flash 64, München, Germany) at our hospital. The average HU values were measured in the cancellous bone of the frontal skull using the “linear histogram graph” function of the picture archiving and communication system (PACS) at our hospital (Fig 1)

The PACS automatically calculates and provides maximum, minimum, and average HU values according to the drawing line. For measurement of HU values in the frontal skull, we used the axial CT where the lateral ventricles immediately disappear or one or two slices above or below. To reduce variations in HU values due to regional heterogeneity of skull HU, we set a relative constant location for the HU measurement. We think that the CT cut level where the lateral ventricles immediately disappear is appropriate for HU measurement because it was easy to locate and exhibited relatively thick cancellous bone in the frontal skull. All brain CT images on the bone setting were magnified for the measurement of HU values in the cancellous bone of the frontal skull to avoid including cortical bone, especially in patients with narrow intercortical space of the frontal skull. Average HU values were measured and recorded in all patients at four locations in the frontal bone, to minimize measurement errors. We drew four lines along the cancellous bone of the frontal skull between left and right coronal sutures (Fig 1). All radiological evaluations were conducted by two faculty neurosurgeons who were blinded to the clinical data of all patients.

### Statistical methods

All patients were classified into normal, osteopenic, or osteoporotic groups based on the BMD T-scores. Continuous variables were expressed as mean ± SD or median with interquartile range, while discrete variables were expressed as a number with a percentage. The chi-square test for discrete variables and one-way analysis of variance (ANOVA) for continuous variables were used to assess differences between BMD categories.

Box-plots with jittering were used to visualize associations between age and frontal skull HU classified by sex. We generated a scatter-plot with a regression line or a line determined by locally weighted scatter-plot smoothing (LOWESS) to graphically represent associations between T-score and average HU of the frontal skull. The average frontal skull HU used for analysis was defined as the average of the four HU values of the frontal bone.

A receiver operating characteristic (ROC) curve was generated, and the area under the (AUC) was used to determine the HU values of the frontal skull for predicting osteoporosis. The value of the frontal skull HU that showed the highest sensitivity and specificity was considered the optimal cutoff value. *p* < 0.05 was considered statistically significant. All statistical analyses were performed using R version 3.3.3 (https://www.r-project.org/).

## Results

### Patient characteristics

We enrolled 899 patients who underwent one or more DXA and brain CT scans with an interval of < 1 year between DXA and brain CT at our hospital from 1 January 2010 to 31 December 2016. The mean age of the patients was 67.5 years, and 81.6% were female. There were significant differences in mean skull HU values among patients in different BMD categories. Descriptive data are shown in Tables 1 and 2.

**Table 1:** Characteristics of the study patients.

Characteristics	Normal (*n* = 188)	Osteopenia (*n* = 352)	Osteoporosis (*n* = 359)	Total (*n* = 899)	*p*
Sex					< 0.001
Female, *n* (%)	128 (68.1)	292 (83.0)	314 (87.5)	734 (81.6)	
Age, mean ± SD, years	61.7 ± 11.4	65.8 ± 11.4	72.2 ± 10.4	67.5 ± 11.7	< 0.001
Age-group, *n* (%), years					< 0.001
< 65	105 (55.9)	153 (43.5)	79 (22.0)	337 (37.5)	
≧ 65	83 (44.1)	199 (56.5)	280 (78.0)	562 (62.5)	
Age-group, *n* (%), years					< 0.001
19–49	26 (13.8)	22 (6.2)	6 (1.7)	54 (6.0)	
50–59	56 (29.8)	80 (22.7)	42 (11.7)	178 (19.8)	
60–69	48 (25.5)	105 (29.8)	77 (21.4)	230 (25.6)	
70–79	50 (26.6)	111 (31.5)	147 (40.9)	308 (34.3)	
80–99	8 (4.3)	34 (9.7)	87 (24.2)	129 (14.3)	
T-score, mean ± SD	-0.3 ± 0.6	-1.8 ± 0.4	-3.3 ± 0.6	-2.1 ± 1.3	< 0.001
Lumbar spine	0.1 ± 0.9	-1.4 ± 1.0	-3.0 ± 0.9	-1.7 ± 1.5	< 0.001
Femoral neck	-0.0 ± 0.7	-1.2 ± 0.8	-2.5 ± 1.0	-1.5 ± 1.3	< 0.001

SD, standard deviation

**Table 2:** HU values based on BMD categories and patient characteristics.

HU values, mean ± SD					
Variables	Normal (*n* = 188)	Osteopenia (*n* = 352)	Osteoporosis (*n* = 359)	Total (*n* = 899)	*p*
Location					
Overall	843.6 ± 203.4	660.8 ± 193.0	515.1 ± 177.5	640.8 ± 225.5	< 0.001
Right lateral	781.8 ± 207.9	608.5 ± 187.5	474.4 ± 162.5	591.2 ± 215.5	< 0.001
Right medial	893.9 ± 224.1	707.5 ± 221.0	547.7 ± 202.3	682.7 ± 250.4	< 0.001
Left medial	907.5 ± 229.6	705.5 ± 225.6	544.5 ± 204.4	683.5 ± 256.8	< 0.001
Left lateral	791.2 ± 213.9	621.8 ± 184.3	493.5 ± 177.9	606.0 ± 218.5	< 0.001
Average, medial	900.7 ± 219.1	706.5 ± 219.0	546.1 ± 199.3	683.1 ± 249.4	< 0.001
Average, lateral	786.5 ± 202.3	615.2 ± 180.5	484.0 ± 165.1	598.6 ± 211.9	< 0.001
Sex					
Male	811.8 ± 187.2	679.6 ± 189.6	561.8 ± 189.0	695.6 ± 212.3	< 0.001
Female	858.5 ± 209.6	657.0 ± 193.8	508.4 ± 175.1	628.5 ± 226.6	< 0.001
Age-group, years					
< 65	877.3 ± 208.2	708.8 ± 196.4	614.2 ± 193.6	739.1 ± 222.7	< 0.001
≧ 65	801.1 ± 189.9	624.0 ± 182.5	487.1 ± 162.5	581.9 ± 205.9	< 0.001
Age-group, years					
19–49	885.0 ± 223.7	795.0 ± 180.0	822.7 ± 142.3	841.4 ± 200.6	0.298
50–59	910.9 ± 197.9	726.7 ± 185.3	628.6 ± 205.1	761.5 ± 221.5	< 0.001
60–69	817.7 ± 175.3	642.3 ± 173.0	538.9 ± 164.6	644.3 ± 197.3	< 0.001
70–79	791.1 ± 207.6	623.0 ± 206.7	482.8 ± 158.1	583.4 ± 215.6	< 0.001
80–99	722.0 ± 163.2	599.8 ± 156.4	472.4 ± 163.0	521.4 ± 177.2	< 0.001

HU, Hounsfield units; BMD, bone mineral density; SD, standard deviation

### Associations between skull HU and age

We observed a decrease in skull HU values of the frontal bone with increasing age. The skull HU values showed significant differences between age-groups (*p* < 0.001; S2 Fig A). When we divided the patients by sex, there was a significant negative correlation between skull HU and age-group in female subjects (*p* < 0.001; S2 Fig B), but age-group was not significantly associated with frontal skull HU in male subjects (*p* = 0.996).

### Associations between skull HU and T-score

**Fig 2:**
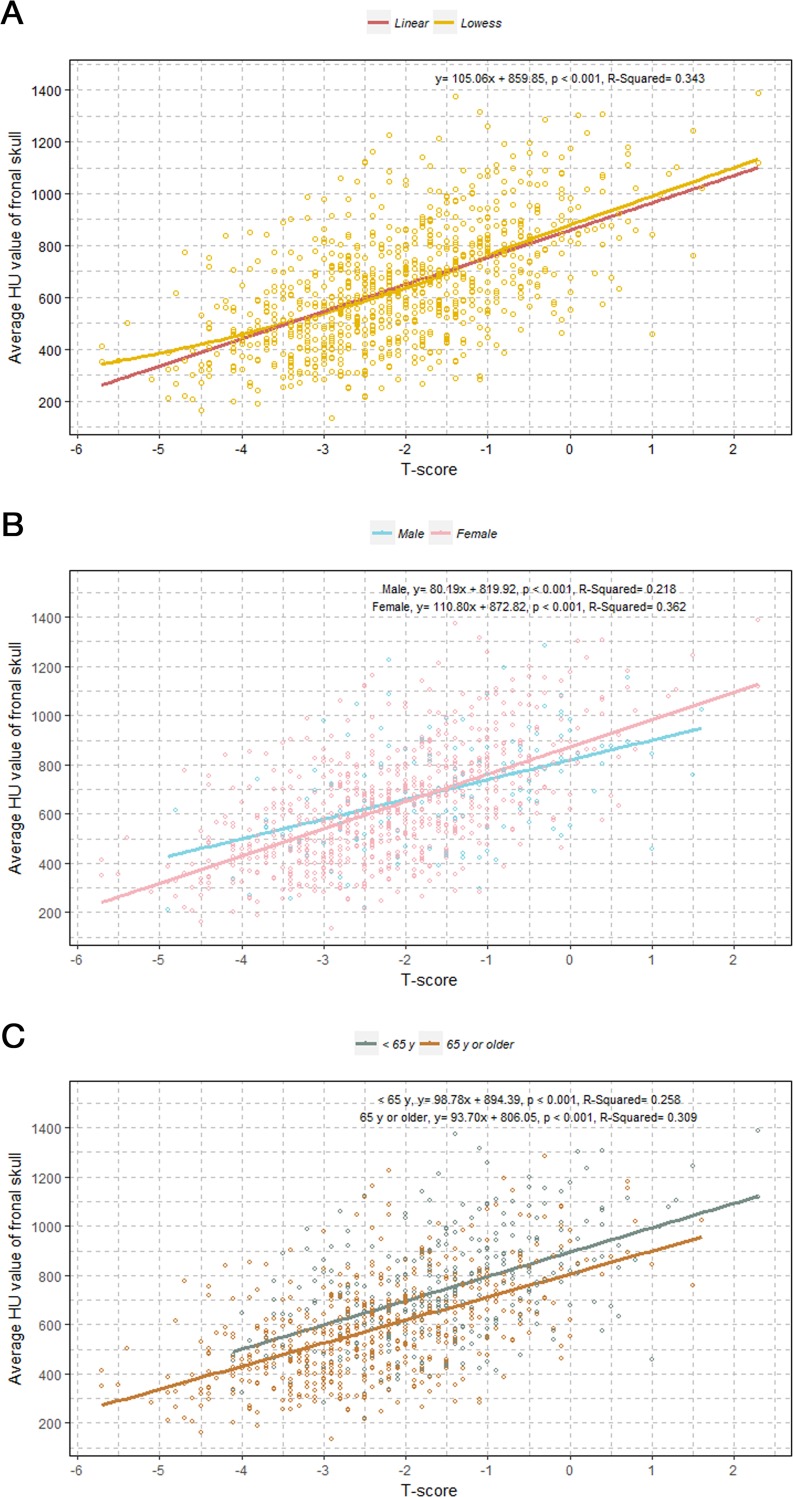
Scatter-plots with linear regression lines depicting the associations between T-scores and HU values of the frontal skull. (A) LOWESS lines showing the associations between T-score and skull HU in all study patients; (B) Linear lines showing the associations between T-score and skull HU classified by sex; (C) Linear lines showing the associations between T-score and skull HU classified by age-group.

We observed an increase of approximately 105 skull HU per T-score increase of 1 with approximately 34% explanatory power (β = 105.06, *p* < 0.001, R^2^ = 0.343; Fig 2A)

**Fig 3:**
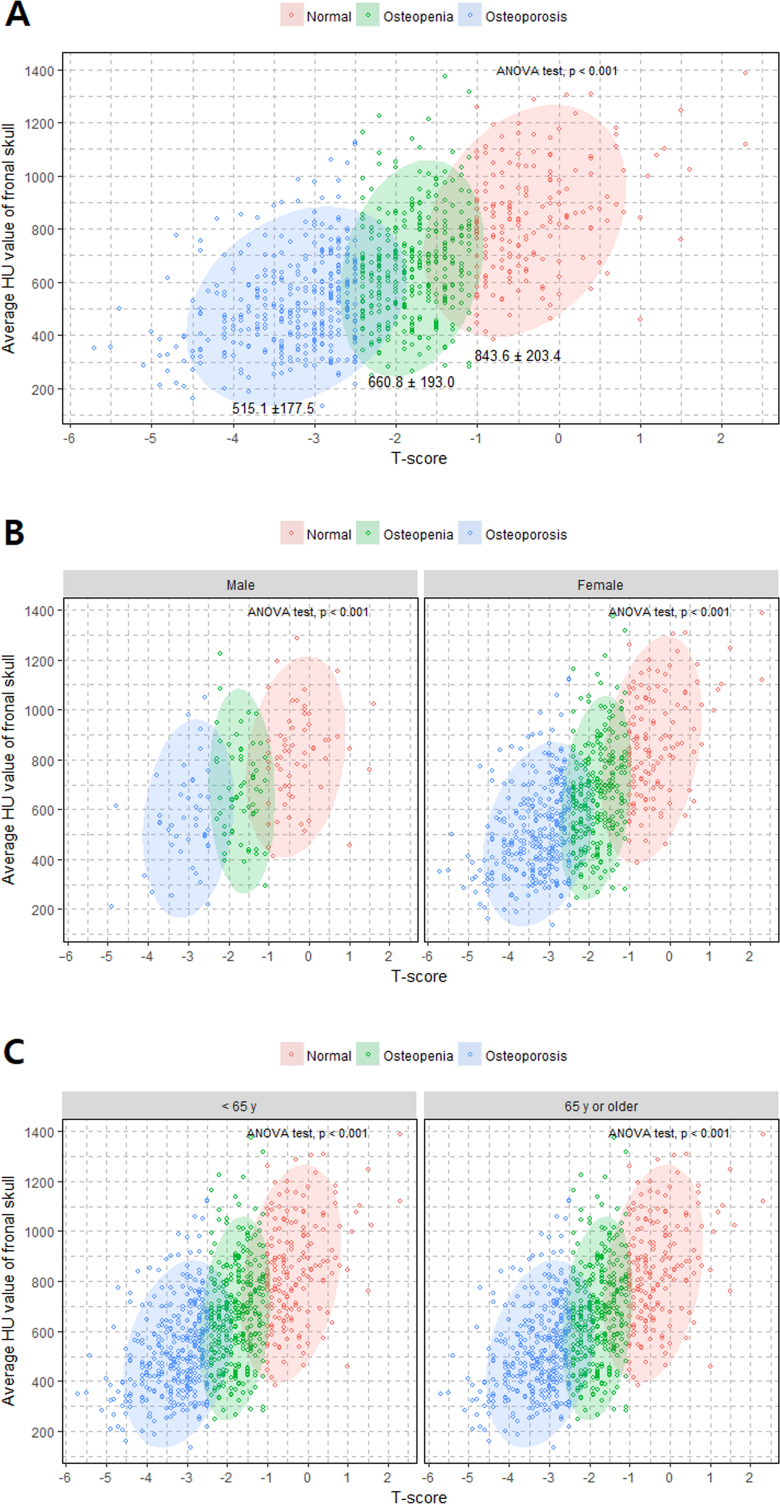
Cluster plots showing the distributions of skull HU values based on BMD categories. (A) All study patients. (B) Classified by sex. (C) Classified by age group.

When we divided the patients by sex, female subjects yielded a steeper slope (β = 110.80, *p* < 0.001) than male subjects (β = 80.19, *p* < 0.001) (Fig 2B). However, the < 65 years age-group and the ≧ 65 years age-group showed similar slopes between skull HU and T-score (β = 98.78 in the younger age-group vs. β = 93.70 in the older age-group), with overall higher skull HU values in the younger age group (Fig 2C). The associations between each of the four HU values of the frontal bone and T-score are shown in S3 Fig. A cluster-plot showing significant differences in skull HU values between the three BMD groups is shown in Fig 3A.

Mean frontal skull HUs were 515.1 ± 177.5 (SD) in the osteoporosis group, 660.8 ± 193.0 in the osteopenia group, and 843.6 ± 203.4 in the normal BMD group (*p* < 0.001, ANOVA). When we separated the patients according to sex and age, the differences between the three BMD groups remained statistically significant (Fig 3B and 3C). The female group yielded a lower overall mean skull HU value than the male group (628.5 vs. 695.6), and the aged ≧ 65 years group yielded a lower overall mean skull HU value than the aged < 65 years group (581.9 vs. 739.1) (Table 2).

**Fig 4:**
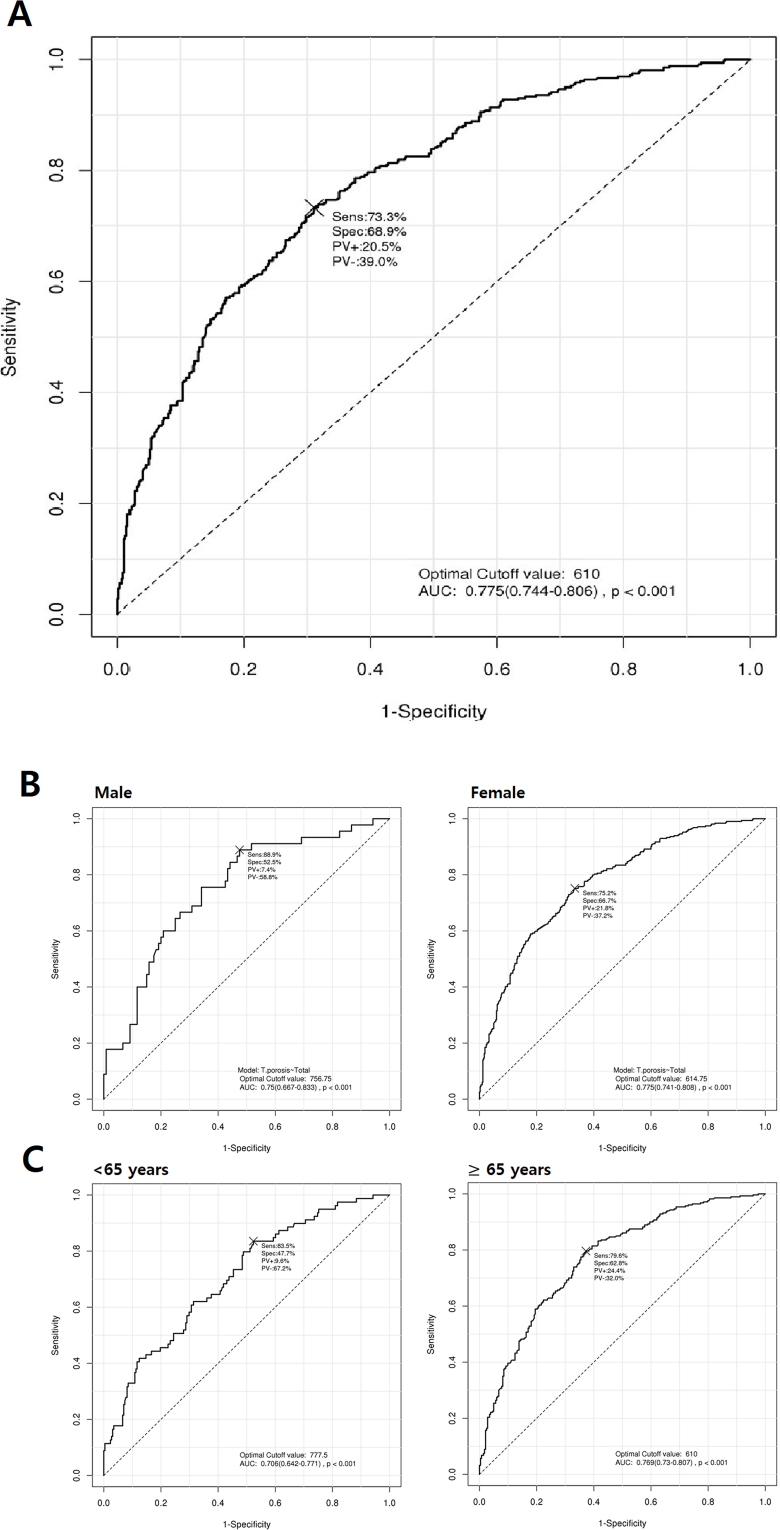
ROC curves for the determination of optimal cutoff values for predicting osteoporosis. (A) All study patients. (B) Classified by sex. (C) Classified by age group.

According to the ROC curve used to assess skull HU threshold for identifying osteoporosis, the optimal cutoff value for the prediction of osteoporosis was 610.0 HU (AUC = 0.775, 95% CI 0.744–0.806, *p* < 0.001) based on all patients (Fig 4A).

We also performed ROC curve analyses classified by sex and age. The respective threshold values for male and female subjects were 756.8 HU (AUC = 0.750, 95% CI 0.667–0.833, *p* < 0.001) and 614.8 HU (AUC = 0.775, 95% CI 0.741–0.808, *p* < 0.001) (Fig 4B). The respective threshold values for the aged < 65 years and aged ≥ 65 years groups were 777.5 HU (AUC = 0.706, 95% CI 0.642–0.771, *p* < 0.001) and 610.0 HU (AUC = 0.769, 95% CI 0.730–0.807, *p* < 0.001) (Fig 4C).

## Discussion

In the current study, average frontal skull HU values were significantly correlated with systemic BMD. Overall, there was an increase of approximately 105 HU in the frontal skull per T-score increase of 1, and a skull HU threshold for osteoporosis of approximately 610. Female subjects yielded lower HU cutoff values for the prediction of osteoporosis than male subjects, and subjects aged ≥ 65 years yielded lower HU cutoff values than those aged < 65 years. To the best of our knowledge, this study is the first to evaluate possible connections between skull HU values and T-scores in the lumbar spine or femoral neck.

The CT-derived values (measured in HU) assigned to each pixel represent the average linear attenuation coefficient of the corresponding voxel, and the values are calculated using the formula HU = (1000 × [μ_voxel_− μ_water_]) / μ_water_ [10,11]. The reconstructed pixel values reflect relative linear attenuation coefficients whose CT numbers can be compared using a CT number scale in which –1000 represents the attenuation of air, and 0 is the attenuation of water, with no upper limit [10]. A previous study showed that the HU of the vertebral body (cancellous bone) represents an average of the linear attenuation coefficients of the mineral, collagen, soft tissue, water, and fat in the vertebral body [12]. We hypothesized that the HU of the cancellous portion of the skull bone may also represent meaningful linear attenuation coefficients similar to the vertebral body.

DXA has been widely used for the diagnosis of osteoporosis. However, previous studies indicate that the majority of patients at high-risk of osteoporosis have not been adequately evaluated for BMD [13,14]. Therefore, various alternative tools for osteoporosis screening have been suggested. Previous studies have reported positive correlations between T-scores and HU values measured from the trabecular portions of several specific sites, including cervical or lumbar spine, distal ulnar, wrist capitate bone, and mandibular bone [13–21]. Schreiber et al. [17] showed that HU value in the lumbar vertebral body was associated with systemic BMD (BMD and T-score) and suggested that CT scans of the spine may represent an alternative screening method for detecting osteoporosis. Another study indicated that HU values in the humerus were correlated with femoral neck BMD and T-score [22]. Other studies have identified HU cutoff values in several anatomical sites for the prediction of osteoporosis using ROC analysis. In a recent study [13], lower HU values of the distal ulnar were significantly associated with low BMD, with a high degree of sensitivity and negative predictive value. Pickhardt et al. [23] found that a maximum threshold value of 135 HU at the L1 vertebral body yielded a good balance between sensitivity and specificity with regard to distinguishing osteoporosis from osteopenia and normal BMD. Because HU values differ depending on anatomical site, we think that identifying HU values for the prediction of osteoporosis at specific anatomical sites will prove beneficial. The prediction of osteoporosis based on HU values derived from CT scans at various anatomical sites may be helpful for identifying patients who require further evaluation or prevention of osteoporosis by clinical physicians of various types. In the neurological outpatient department for example, brain CT scans are routinely performed in patients with minor head trauma, headache, and syncope, among others. Therefore, convenient prediction of bone quality may be possible by measuring HU values using brain CT scans, especially among patients at higher risk for osteoporosis such as menopausal or postmenopausal women. In addition, measuring HU values via brain CT may require no additional cost, equipment, or patient time [21,23]. Screening for osteoporosis by opportunistically measuring HU values via brain CT may be helpful for detecting osteoporosis in patients and reducing fracture risk through subsequent appropriate evaluation and treatment.

The current study had some limitations. First, due to the retrospective nature of the study CT scans and DXA measurements were not performed at the same time. Although we only included patients who underwent these procedures less than 1 year apart, heterogeneity of the time interval may have affected the results. Second, anti-osteoporotic medication may also have affected the results. However, we included the lowest T-score from each patient who underwent DXA more than once to reduce the effects of anti-osteoporotic medications in this study. Third, because all CT scans were performed at a single institution with a single CT scanner, it is difficult to generalize the study’s HU values. However, a previous study derived HU values from nine tissue types using five CT scanners, and variations in HU values between the five scanners were in the range of 0–20 HU [24]. Therefore, while our skull HU values are not absolute, they are likely representative. Fourth, in some patients it was not possible to measure HU values via brain CT. We could not measure HU values in the frontal bone in 32 of 931 patients (3.4%) due to narrow or absent intercortical space.

## Conclusions

Despite the above-described limitations, we sought to evaluate associations between HU values of the frontal skull and T-scores at the lumbar spine or femoral neck, and we detected a significant positive relationship between skull HU and systemic BMD. Therefore, we suggest that clinical brain CT scans may provide an opportunity to detect osteoporosis, and patients with values of < 610 HU as determined via brain CT may be considered for further evaluation for possible osteoporosis.

## Supporting information

S1 Fig The study included 899 patients with DXA and brain CT performed < 1 year apart. (TIF)

S2 Fig Box-plots showing associations between age and skull HU values. (A) All patients. (B) Classified by sex.
(TIF)

S3 Fig Scatter-plot with lines showing positive associations between T-score and each of the four HU values of the frontal bone. HU = Hounsfield units.
(TIF)
