# Supplementary figures and images for: Opportunistic osteoporosis screening via the measurement of frontal skull Hounsfield units derived from brain computed tomography images
Source: PLoS One. 2018 May 10;13(5):e0197336. doi: 10.1371/journal.pone.0197336 (PMC5945032; doi:10.1371/journal.pone.0197336)

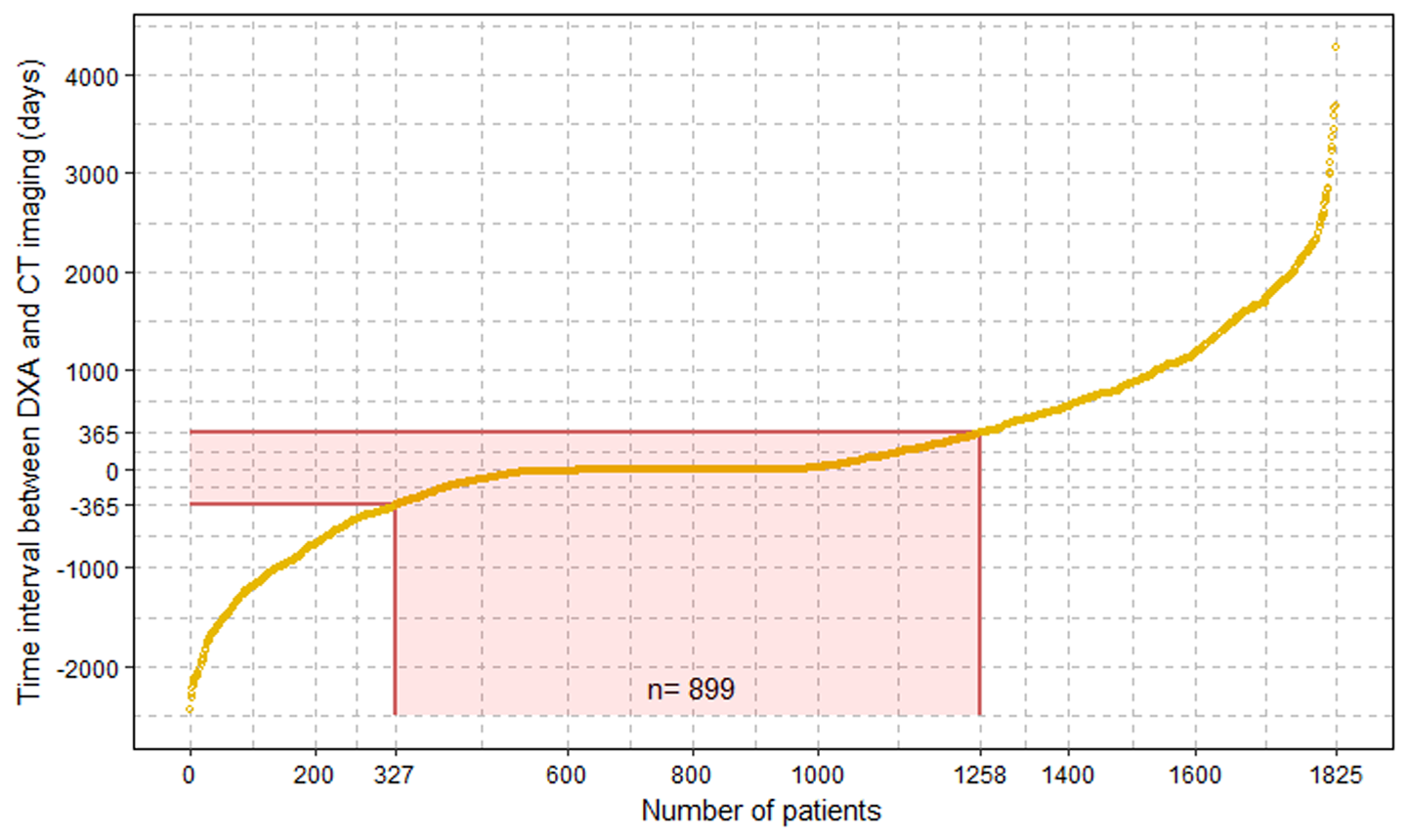

Supplement: S1 Fig — (TIF) [file pone.0197336.s001.tif]

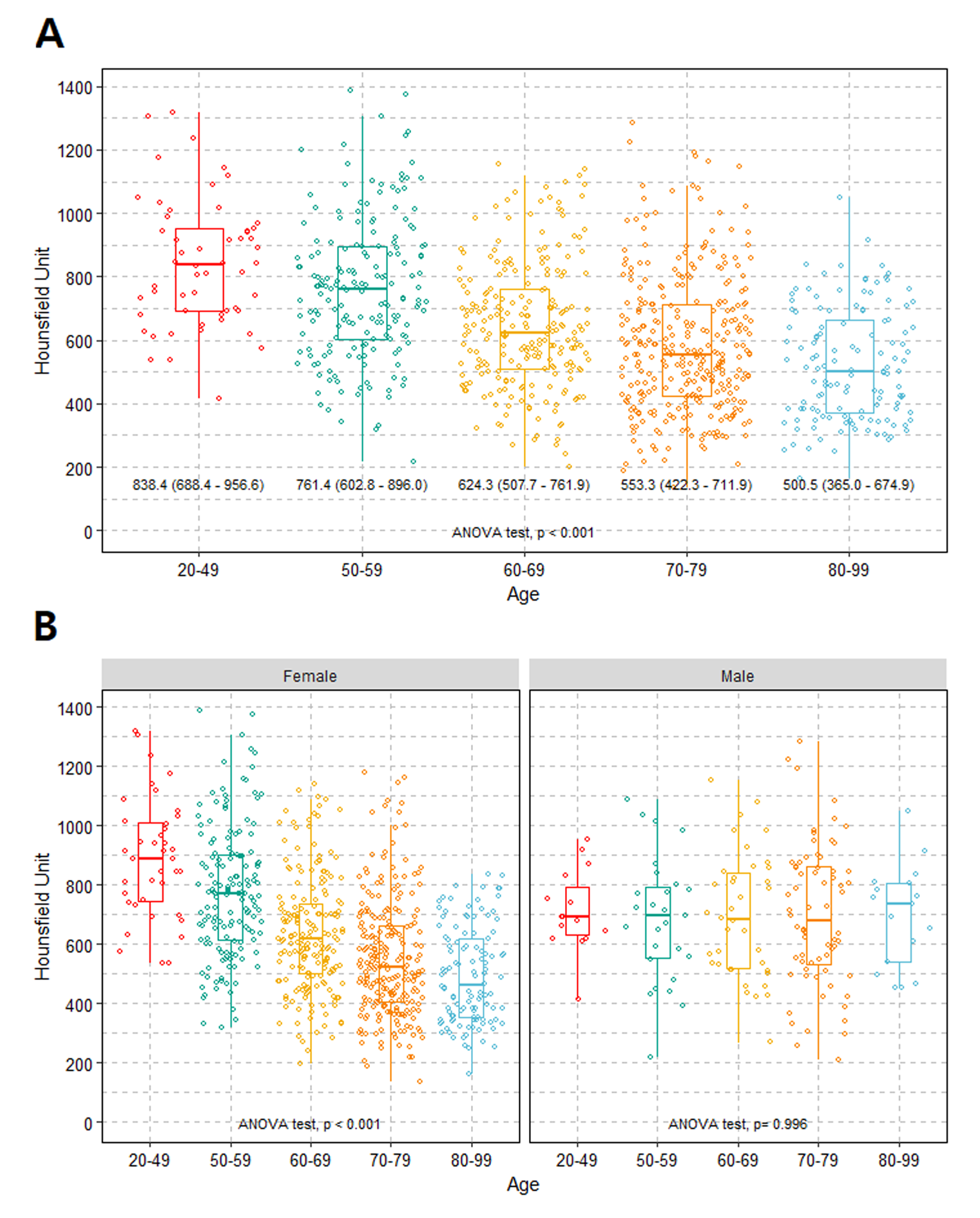

Supplement: S2 Fig — (A) All patients. (B) Classified by sex. (TIF) [file pone.0197336.s002.tif]

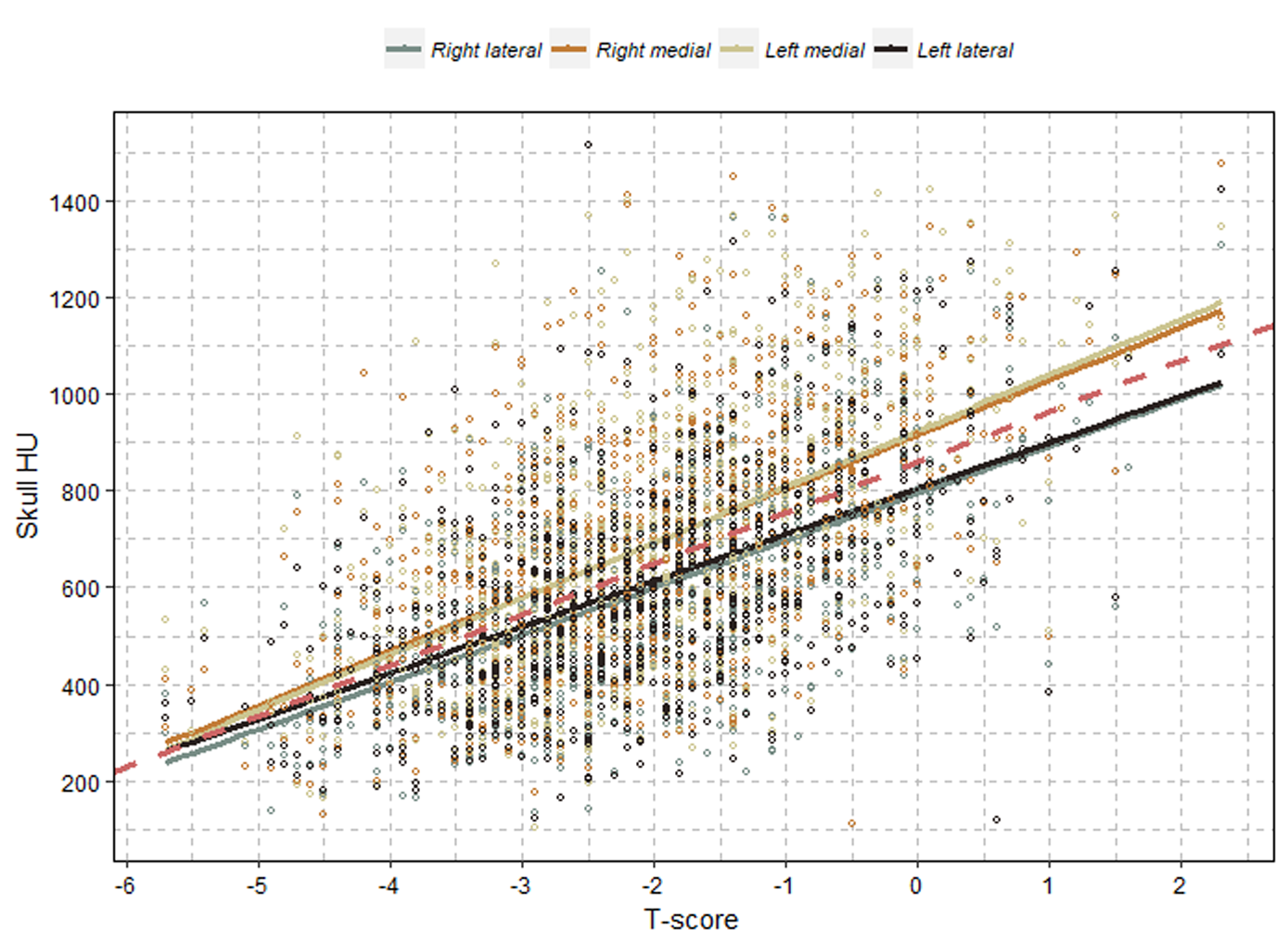

Supplement: S3 Fig — HU = Hounsfield units. (TIF) [file pone.0197336.s003.tif]
